# Prognosis and nomogram for predicting postoperative survival of duodenal adenocarcinoma: A retrospective study in China and the SEER database

**DOI:** 10.1038/s41598-018-26145-6

**Published:** 2018-05-21

**Authors:** Sujing Jiang, Rongjie Zhao, Yiran Li, Xufeng Han, Zhen Liu, Weiting Ge, Ying Dong, Weidong Han

**Affiliations:** 1 Department of Medical Oncology, The Second Affiliated Hospital, College of Medicine, Zhejiang University, Hangzhou, Zhejiang, China; 2 Department of Medical Oncology, Sir Run Run Shaw Hospital, College of Medicine, Zhejiang University, Hangzhou, Zhejiang, China; 3 Department of Internal Medicine, Yuyao Traditional Chinese Medicine Hospital, Yuyao, Zhejiang, China; 4 Cancer Institute, The Second Affiliated Hospital, College of Medicine, Zhejiang University, Hangzhou, Zhejiang, China

## Abstract

As primary duodenal adenocarcinoma is rare, the prognostic factors of this disease remain insufficiently explored, especially in China. We identified postoperative duodenal adenocarcinoma patients at a Chinese double-center (from 2006 to 2016) or who were registered with the Surveillance, Epidemiology, and End Results (SEER) database (from 2004 to 2014). Clinicopathological features and significant prognostic factors for cancer-specific survival (CSS) were reviewed and analyzed by using univariate and multivariate Cox proportional hazards regression. Then, a nomogram predicting CSS was constructed based on the SEER database and validated externally by using the separate Chinese cohort. Totally, 137 patients from the Chinese double-center and 698 patients from the SEER database were included for analysis. The multivariate analyses showed that age, tumor grade and TNM stage were independent prognostic factors. The nomogram constructed using these factors showed a clear prognostic superiority to the AJCC-TNM classification, 7^th^ ed. (C-index: SEER cohort, 0.693 vs 0.625, *P* < 0.001; Chinese cohort, 0.677 vs 0.659, *P* < 0.001, respectively). In summary, the valuable prognostic factors in patients with duodenal adenocarcinoma were age, tumor grade and TNM stage. This study developed a nomogram that can precisely predict the CSS for postoperative duodenal adenocarcinoma patients.

## Introduction

Primary duodenal adenocarcinoma is a rare but extremely aggressive tumor that account for less than 0.5% of gastrointestinal malignancy. Although the duodenum constitutes less than 10% of the length of the small intestine, it accounts for approximately 45% of small bowel cancer^1,2^. Diagnosis of duodenal adenocarcinoma is often delayed because the symptoms are nonspecific, with nearly half of patients presenting with abdominal pain. This situation resulted in negative influences on the survival outcome^3,4^. Surgery remains the primary treatment for duodenal adenocarcinoma. Pancreaticoduodenectomy and segmental resection are performed most commonly^5^. However, given the low incidence of duodenal adenocarcinoma and limited number of clinical studies, there is no consensus as to the most effective treatment strategy. The extent of resection for duodenal adenocarcinoma is still controversial^6^. In recent decades, different studies attempted to identify the prognostic factors in patients with duodenal adenocarcinoma. Most studies recognize regional lymph node metastasis as having a negative impact on survival^7–9^. However, prognostic factors such as age, sex, tumor size, tumor grade and T stage are inconsistently associated with outcomes, and such discrepancies are likely due to small sample sizes^10^.

In this study, based on data from the Chinese double-center and the Surveillance, Epidemiology, and End Results (SEER) database, we aimed to retrospectively analyze and identify the clinicopathologic features and independent prognostic factors of postoperative patients with duodenal adenocarcinoma. Then, we divided patients in the SEER database into the train cohort and test cohort. Based on the train cohort, we constructed a nomogram by combining clinicopathologic variables and compared their prognostic value with that of the AJCC-TNM classification, 7^th^ ed. In addition, we used the independent Chinese cohort for external validation.

## Results

### Patient characteristics

In total, 137 patients met the eligibility criteria in the Chinese cohort, 84 (61.3%) were male, and 53 (38.7%) were female. Overall, 698 postoperative patients with duodenal adenocarcinoma in the SEER database were eligible, including 352 (50.4%) male and 346 (49.6%) female patients (Table 1).

**Table 1:** Clinicopathological features of the Chinese and SEER cohort with postoperative duodenal adenocarcinoma.

Variable	Chinese cohort	SEER cohort
Sex		
Men	84 (61.3%)	352 (50.4%)
Female	53 (38.7%)	346 (49.6%)
Age		
≤58	55 (40.1%)	238 (34.1%)
59–75	58 (42.3%)	315 (45.1%)
>75	24 (17.8%)	145 (20.8%)
Race		
Caucasians	—	532 (76.2%)
African Americans	—	112 (16.0%)
Others	—	54 (7.7%)
Tumor grade		
Well	41 (29.9%)	55 (7.9%)
Moderate	68 (49.6%)	365 (52.3%)
Poor	28 (20.4%)	264 (37.8%)
Undifferentiated		14 (2.0%)
Size		
≤2 cm	59 (43.1%)	86 (12.3%)
2–4 cm	54 (39.4%)	308 (44.1%)
>4 cm	24 (17.5%)	304 (43.65)
T stage		
T1	11 (8.9%)	33 (4.7%)
T2	35 (25.5%)	58 (8.3%)
T3	32 (23.4%)	254 (36.4%)
T4	59 (43.1%)	353 (50.6%)
N stage		
N0	95 (69.3%)	269 (38.5%)
N1	32 (23.4%)	255 (36.5%)
N2	10 (7.3%)	174 (24.9%)
M stage		
M0	121 (88.3%)	650 (93.1%)
M1	16 (11.7%)	48 (6.9%)
Stage		
Stage I	36 (26.3%)	62 (8.9%)
Stage II	49 (35.8%)	201 (28.8%)
Stage III	36 (26.3%)	387 (55.4%)
Stage IV	16 (11.7%)	48 (6.9%)
Vascular invasion		
No	112 (81.8%)	—
Yes	25 (18.2%)	—
Perineural invasion		
No	113 (82.5%)	—
Yes	24 (17.5%)	—

The median patient age was 64 years (range, 35–94 years) in the Chinese cohort and 66 years (range, 22–94 years) in the SEER cohort. The median follow-up for the survivors was 26 months (range, 1–130 months) in the Chinese cohort compared with 20.5 months (range, 1–131 months) in the SEER cohort. During the follow-up period, 49 out of 137 (35.8%) patients died of cancer-associated death in the Chinese cohort compared with 308 out of 698 (44.1%) patients in the SEER cohort. The 3- and 5-year cancer-specific survival (CSS) values were 67% and 39% in the Chinese cohort and 52% and 38% in the SEER cohort, respectively.

Histopathologically, most patients had moderately differentiated tumors in the Chinese and SEER cohort (n = 68, 49.6% vs n = 365, 52.3%). However, patients with duodenal adenocarcinoma tended to present with more poorly differentiated tumors in the SEER cohort than those  in the Chinese cohort (n = 264, 37.8% vs n = 28, 20.4%). According to the AJCC-TNM classification, 7^th^ ed., patients in the SEER cohort were approximately twice as likely to be diagnosed as having stage III cancer than those in the Chinese cohort (55.4% vs 26.3%). Conversely, the percentage of patients with stage I cancer in the Chinese cohort was significantly higher than that of patients in the SEER cohort (26.3% vs 8.9%). The majority of the patients had pathologic T4 cancer in both the Chinese and SEER cohorts. Patients in the SEER cohort were more likely to present with LN metastasis than those in the Chinese cohort (61.4% vs 30.7%). Distant metastasis was found in 16 patients (11.7%, including 9 patients with liver metastases, 4 with omentum metastases, and 3 with abdominal wall metastases) in the Chinese cohort vs 48 patients (6.9%) in the SEER cohort. In addition, tumor vascular and perineural invasion occurred in 18.2% and 17.5% of the Chinese cohort, respectively.

Of the patients in the Chinese cohort, 90 patients (65.7%) suffered from alimentary symptoms, including 53 (38.7%) patients with abdominal pain, 25 (18.2%) patients with nausea and vomiting, and 12 (8.8%) patients with gastrointestinal bleeding. Jaundice and itching were documented in 32 (23.4%) and 11 (8.0%) patients, respectively. A total of 8 patients (13.1%) were asymptomatic at diagnosis. Thirty-four patients presented with other symptoms, such as weight loss, fatigue and fever. Serum tumor markers were elevated in 110 patients before surgery; 36.4% and 16.4% of patients had abnormal elevation of carbohydrate antigen 19–9 (CA19-9) and carcinoembryonic antigen (CEA), respectively (Table 2).

**Table 2:** Incidence of initial symptoms and laboratory tests in Chinese cohort with postoperative duodenal adenocarcinoma.

Variable	Patient demographics (%)
Symptom at diagnosis	
Alimentray symptoms	90 (65.7%)
Abdominal pain	53 (38.7%)
Nausea and vomiting	25 (18.2%)
Gastrointestional bleeding	12 (8.8%)
Jaundice	32 (23.4%)
Itching	11 (8.0%)
Asymoptomatic	8 (5.8%)
Others*	34 (24.8%)
CEA	
Normal	70 (67.2%)
High	18 (13.1%)
Inconclusion	27 (19.7%)
CA19–9	
Normal	70 (51.1%)
High	40 (29.2%)
Inconclusion	27 (19.7%)

Abbreviations: CEA, carcinoembryonic antigen; CA19–9, carbohydrate antigen 19–9; Others* included weight loss, fatigue and fever.

### Univariate and multivariate analyses of CSS prognostic factors

**Figure 1:**
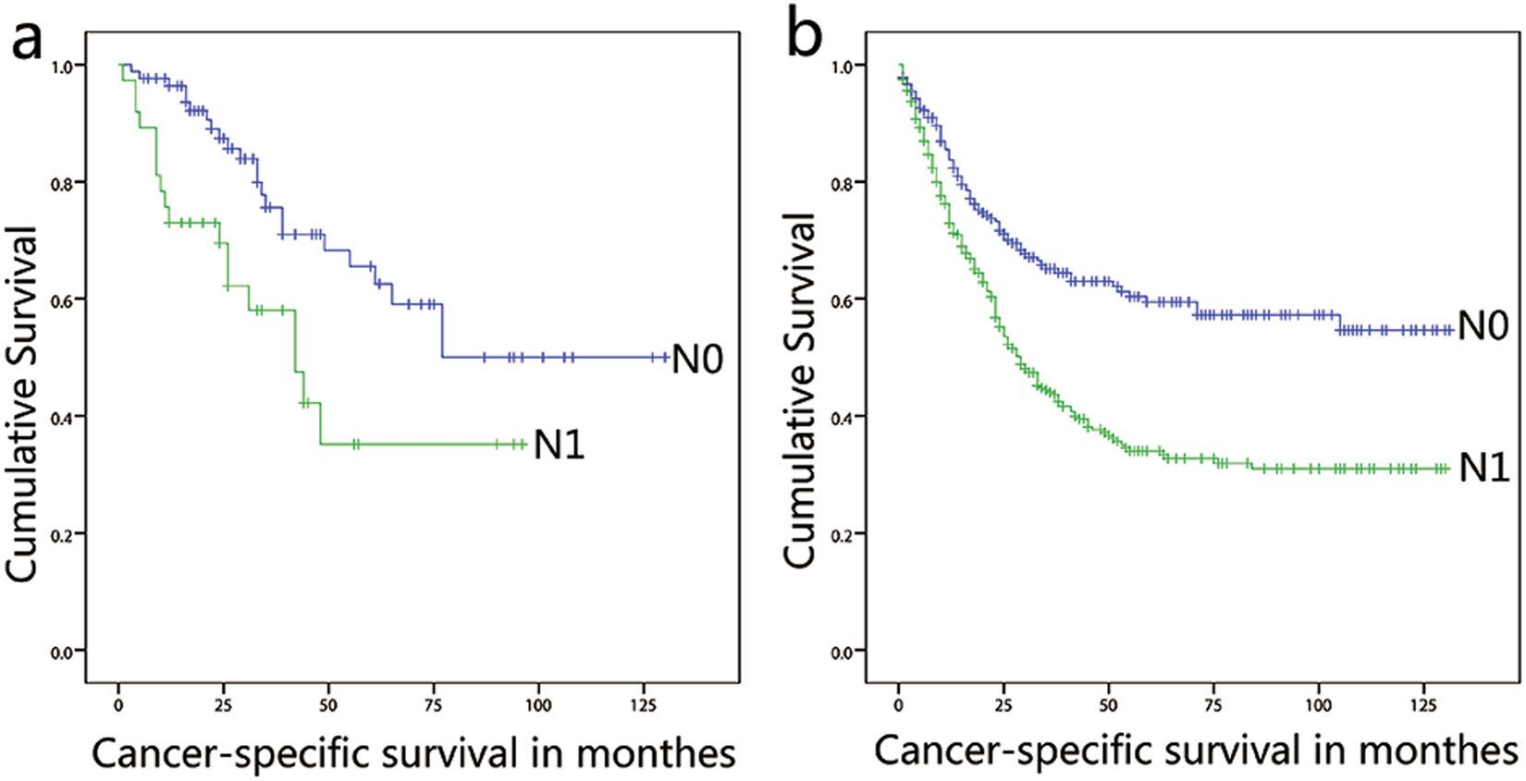
Kaplan-Meier curves of CSS for patients with postoperative duodenal adenocarcinoma in the Chinese cohort (**a**) and SEER cohort (**b**) by risk group stratification within each pN stage.

In the univariate analysis, age, tumor grade, tumor size and TNM category were closely related to CSS in the SEER cohort, but only LN and distant metastasis was associated with CSS in the Chinese cohort (Table 3). The multivariate analyses identified five variables, including older age, advanced grade and TNM category, to be significantly associated with CSS in the SEER cohort. However, in the Chinese cohort, only LN and distant metastasis was statistically significantly different in the CSS (Table 4). The median CSS of the patients with a negative LN was 77 months compared with 48 months in the patients with N1 stage disease (HR: 1.392; 95% CI: 0.708 to 2.734; *P* = 0.337) and 12 months in patients with N2 stage disease (HR: 6.306; 95% CI: 2.776 to 14.321; *P* < 0.001) in the Chinese cohort. In the SEER cohort, the median CSS was 35 months in patients with N1 stage disease (HR: 1.676; 95% CI: 1.273 to 2.206; *P* < 0.001) and 23 months in patients with N2 stage disease (HR: 2.339; 95% CI: 1.744 to 3.137; *P* < 0.001). Kaplan-Meier curves stratified by LN metastasis for both cohorts are shown in Fig. 1.

**Table 3:** Univariate analysis of patients with postoperative duodenal adenocarcinoma in Chinese and SEER cohort.

Variable	Chinese cohort		SEER cohort	
	Hazard Ratio (95%CI)	*P*	Hazard Ratio (95%CI)	*P*
Race				0.477
Caucasians	—	—		
African Americans	—	—	0.944 (0.690–1.290)	0.716
Others	—	—	0.748 (0.468–1.207)	0.234
Sex		0.299		0.119
Male	—	—	—	—
Female	0.733 (0.407–1.320)	0.301	0.837 (0.669–1.047)	0.120
Age		0.127		<0.001
≤58	—	—	—	—
59–75	1.098 (0.571–2.112)	0.779	1.599 (1.216–2.102)	0.001
>75	2.013 (0.985–4.115)	0.550	2.741 (2.020–3.720)	<0.001
Tumor grade		0.110		0.003
Well	—	—	—	
Moderate	1.309 (0.898–1.907)	0.162	0.853 (0.676–1.077)	0.181
Poor	1.223 (0.773–1.936)	0.389	1.145 (0.903–1.451)	0.264
Undifferentiated	—	—	1.728 (1.066–2.803)	0.027
Size		0.275		0.001
≤2 cm	—	—	—	—
2–4 cm	0.683 (0.372–1.253)	0.218	1.667 (1.145–2.426)	0.008
>4 cm	0.549 (0.225–1.339)	0.187	1.133 (0.769–1.669)	0.527
T stage		0.470		<0.001
T1	—	—	—	—
T2	0.769 (0.257–2.302)	0.639	1.947 (0.694–5.464)	0.205
T3	1.273 (0.452–3.586)	0.648	3.488 (1.420–8.567)	0.006
T4	1.397 (0.525–3.721)	0.503	5.407 (2.223–13.155)	<0.001
N stage		<0.001		<0.001
N0	—	—	—	—
N1	1.392 (0.708–2.734)	0.337	1.676 (1.273–2.206)	<0.001
N2	6.306 (2.776–14.321)	<0.001	2.339 (1.744–3.137)	<0.001
M stage		0.007		<0.001
M0	—	—	—	—
M1	2.769 (1.279–5.985)	0.01	2.163 (1.419–3.138)	<0.001
Stage		0.003		<0.001
Stage I	—		—	
Stage II	0.941 (0.414–2.135)	0.884	2.464 (1.306–4.648)	0.005
Stage III	2.268 (1.065–4.830)	0.034	3.699 (2.014–6.795)	<0.001
Stage IV	3.649 (1.439–9.249)	0.006	6.488 (3.257–12.925)	<0.001
Vascular invasion		0.637		
No	—	—	—	—
Yes	1.201 (0.561–2.575)	0.637	—	—
Perineural invasion		0.998		
No	—	—	—	—
Yes	0.999 (0.423–2.360)	0.998	—	—

**Table 4:** Multivariate analysis of patients with postoperative duodenal adenocarcinoma in Chinese and SEER cohort.

Variable	Chinese cohort		SEER cohort	
	Hazard Ratio (95%CI)	*P*	Hazard Ratio (95%CI)	*P*
Race				0.293
Caucasians	—	—	—	—
African Americans	—	—	1.227 (0.888–1.696)	0.215
Others	—	—	0.817 (0.503–1.329)	0.416
Sex		0.299		
Male	—	—	—	—
Female	0.733 (0.407–1.320)	0.301	0.889 (0.706–1.118)	0.313
Age		0.127		<0.001
≤58	—	—	—	—
59–75	1.098 (0.571–2.112)	0.779	1.710 (1.296–2.257)	<0.001
≥75	2.013 (0.985–4.115)	0.55	3.101 (2.275–4.227)	<0.001
Tumor grade		0.110		0.040
Well	—	—	—	—
Moderate	1.309 (0.898–1.907)	0.162	1.063 (0.634–1.718)	0.817
Poor	1.223 (0.773–1.936)	0.389	1.282 (0.759–2.163)	0.353
Undifferentiation	—	—	2.488 (1.125–5.500)	0.024
Size		0.275		0.053
≤2 cm	—	—	—	—
2–4 cm	0.683 (0.372–1.253)	0.218	1.018 (0.730–1.603)	0.697
>4 cm	0.549 (0.225–1.339)	0.187	0.802 (0.533–1.206)	0.289
T stage		0.470		<0.001
T1	—	—	—	—
T2	0.769 (0.257–2.302)	0.639	2.143 (0.741–6.197)	0.159
T3	1.273 (0.452–3.586)	0.648	3.451 (1.332–8.941)	0.011
T4	1.397 (0.525–3.721)	0.503	4.803 (1.876–12.297)	0.001
N stage		<0.001		0.001
N0	—	—	—	—
N1	1.392 (0.708–2.734)	0.337	1.378 (1.033–1.837)	0.029
N2	6.306 (2.776–14.321)	<0.001	1.834 (1.340–2.510)	<0.001
M stage		0.007		<0.001
M0	—	—	—	—
M1	2.769 (1.279–5.985)	0.01	2.191 (1.490–3.220)	<0.001

### Risk of lymph node metastasis

Patients with stage I to III duodenal adenocarcinoma were grouped according to LN metastasis (Table 5). T-stage classification was independently associated with the increased risk of LN metastasis both  in the Chinese and SEER cohorts (Table 6). Tumor grade is an independent factor of LN metastasis in the SEER cohort but not in the Chinese cohort. The rate of LN metastasis increased with a higher T-stage classification (T1, 2.7%; T2, 10.8%; T3, 24.3%; T4, 62.6% in the Chinese cohort vs T1, 1.3%; T2, 5.7%; T3, 32.5%; T4, 60.6% in the SEER cohort; *P* < 0.001).

**Table 5:** Demographic and clinicopathologic variables of postoperative  duodenal adenocarcinoma patients with or without LN metastasis in Chinese and SEER cohort.

Variable	Chinese cohort		SEER cohort	
	LN negative	LN positive	LN negative	LN positive
Sex				
Men	52 (61.9%)	20 (54.1%)	126 (47.5%)	203 (52.7%)
Female	32 (38.1%)	17 (45.9%)	139 (52.5%)	182 (47.3%)
Age				
≤58	36 (42.9%)	14 (37.8%)	84 (31.7%)	132 (34.3%)
59–75	34 (40.5%)	17 (45.9%)	120 (45.3%)	177 (46.0%)
>75	14 (16.7%)	6 (16.2%)	61 (23.0%)	76 (19.7%)
Tumor grade				
Well	39 (46.4%)	19 (51.4%)	35 (13.2%)	18 (4.7%)
Moderate	16 (19.0%)	10 (27.0%)	139 (52.5%)	204 (53.0%)
Poor	29 (34.5%)	8 (21.6%)	85 (32.1%)	155 (40.3%)
Undifferentiated	—	—	6 (2.3%)	8 (2.1%)
Size				
≤2 cm	81 (96.4%)	34 (91.9%)	50 (18.9%)	32 (8.3%)
2–4 cm	3 (3.6%)	3 (8.1%)	100 (37.7%)	179 (46.5%)
>4 cm	—	—	115 (43.4%)	174 (45.2%)
T stage				
T1	10 (11.9%)	1 (2.7%)	26 (9.8%)	5 (1.3%)
T2	30 (35.7%)	4 (10.8%)	36 (13.6%)	22 (5.7%)
T3	20 (23.8%)	9 (24.3%)	109 (41.1%)	125 (32.5%)
T4	24 (28.6%)	23 (62.6%)	94 (35.5%)	233 (60.5%)

**Table 6:** Multivariable logistic regression for predictors of LN metastasis of postoperative  duodenal adenocarcinoma patients in Chinese and SEER cohort.

Variable	Chinese cohort		SEER cohort	
	Hazard Ratio (95%CI)	*P*	Hazard Ratio (95%CI)	*P*
Tumor grade		0.769		0.044
Well	—	—	—	—
Moderate	1.221 (0.413–3.610)	0.719	2.326 (1.212–4.466)	0.011
Poor	1.574 (0.459–5.397)	0.470	2.640 (1.347–5.174)	0.005
Undifferentiated	—	—	2.009 (0.573–7.042)	0.276
T stage		0.004		<0.001
T1	—	—	—	—
T2	1.333 (0.133–13.368)	0.180	2.601 (0.856–7.900)	0.092
T3	4.500 (0.498–40.654)	0.038	4.652 (1.693–12.781)	0.003
T4	9.583 (1.135–80.942)	0.028	10.199 (3.736–27.844)	<0.001

### Construction and validation of the CSS nomogram

**Figure 2:**
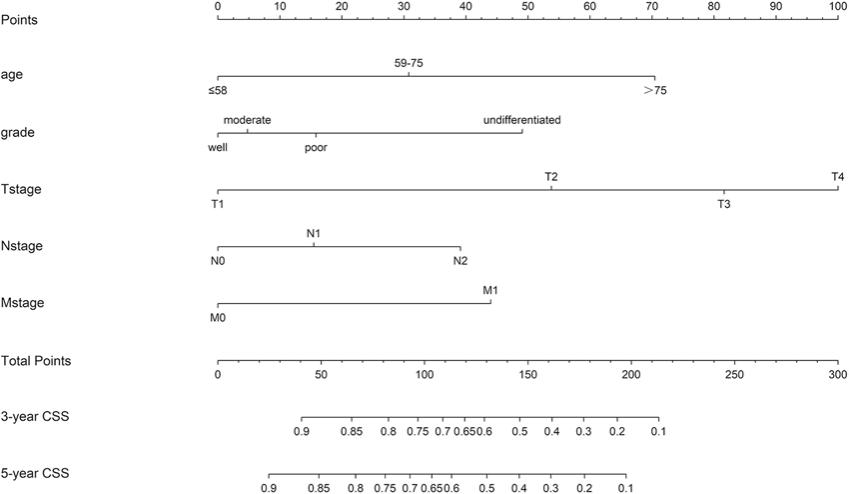
Nomogram predicting the 3-year and 5-year CSS for postoperative  duodenal adenocarcinoma patients.

The TNM-based nomogram, incorporating all the significant independent factors for predicting 3- and 5-year CSS based on the SEER training cohort, was established. Figure 2 shows the prediction of the 3-year and 5-year CSS in the nomogram of the TNM-based model. Each variable was given a score on the points scale. By adding up the total scores shown in the bottom scale, the nomogram could predict the 3-year and 5-year CSS for the individual patients. The C-index for the TNM-based model (0.693; 95% CI, 0.673 to 0.710) was superior to that for the AJCC-TNM classification (0.625; 95% CI, 0.606 to 0.644; *P* < 0.001).

In the test cohort, the C-index of the TNM-based CSS nomogram was (0.681; 95% CI, 0.642 to 0.719) higher than that of the 7th AJCC system (0.634; 95% CI, 0.593 to 0.675; *P* < 0.001). Consistently, in the Chinese external validation cohort, the TNM-based nomogram (0.677; 95% CI, 0.634 to 0.719) still showed superior discrimination compared to the 7th AJCC system (0.659; 95% CI, 0.618 to 0.701; *P* < 0.001).

**Figure 3:**
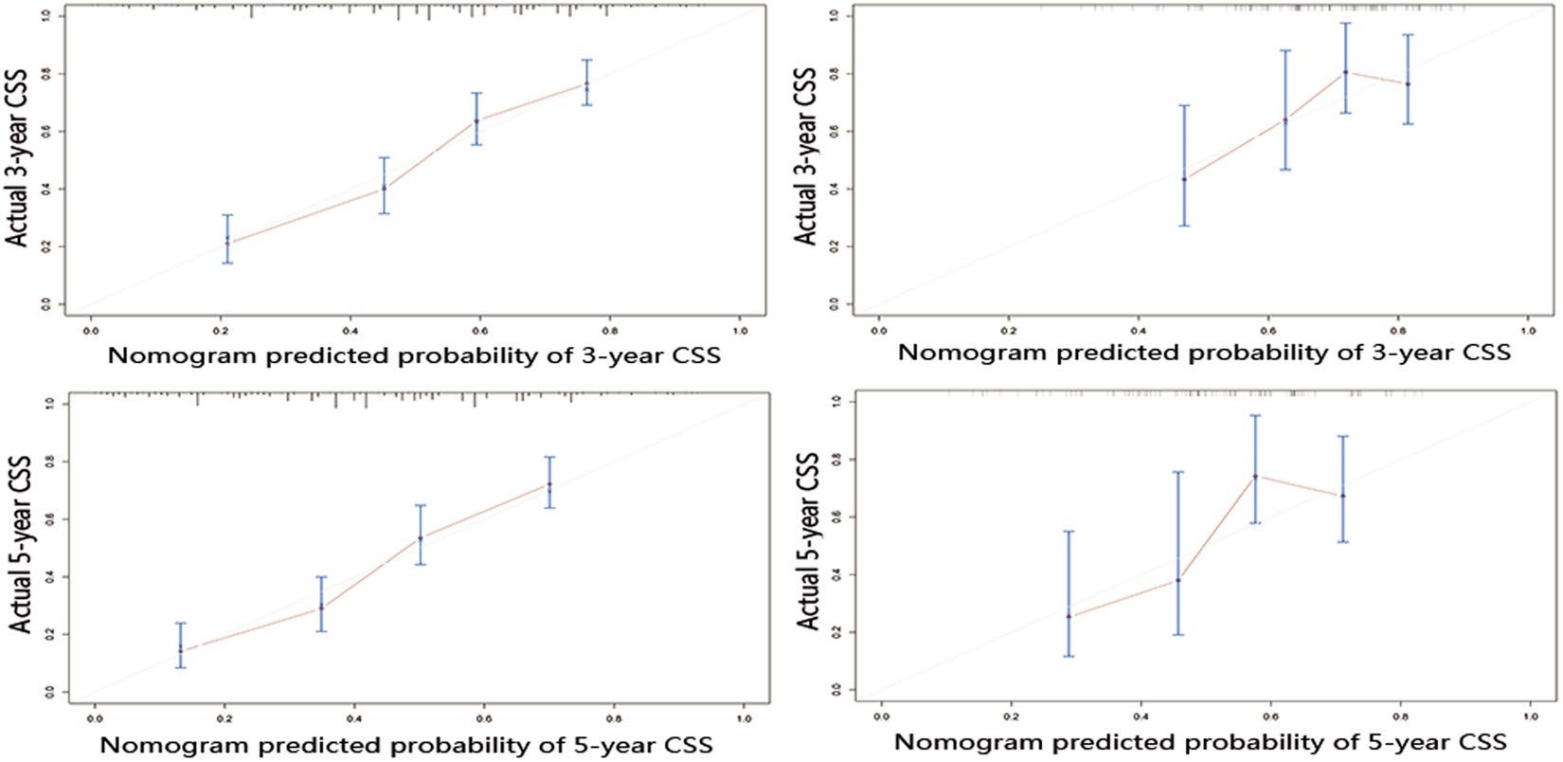
The calibration curves for predicting patient CSS at (**A**) 3-year and (**B**) 5-year in the training cohort, and at (**C**) 3-year and (**D**) 5-year in the external validation cohort. Nomogram-predicted survival is plotted on the x-axis, and the actual survival is plotted on the y-axis.

The calibration plots of the train cohort and the external validation cohort are presented in Fig. 3, which shows the predicted 3- and 5-year CSS probabilities for both the SEER training cohort and the Chinese validation cohort compared with the actual observations.

## Discussion

Duodenal adenocarcinoma is a rare cancer affecting approximately less than 0.5 per 100,000 individuals^5^. Due to the low incidence and prevalence of duodenal adenocarcinoma, few studies have been published, and the relevant survival factors are still controversial^11^. In this retrospective study, we evaluated the clinicopathological characteristics and independent prognostic factors in duodenal adenocarcinoma patients who underwent surgical excision from the Chinese double-center and the SEER database. Independent prognostic factors for CSS were related to age, tumor grade and TNM stage. The TNM-based nomogram included these factors and predicted CSS better than the AJCC TNM staging system, 7^th^ ed.

Several similarities and differences between the Chinese and SEER cohorts were observed in this study. In Chinese cohort, 61.3% were male and 38.7% were female. It is very different from that of the United States (Table 1). Considering this is a retrospective analysis from the double-center, which may result in a selection bias. However, we found our difference of  gender incidence  in small bowel cancers was in line with two studies of small bowel cancer in the Chinese population^12–14^. So there may be difference between the male and female incidence of small bowel cancers in Asian and Caucasians population, and future research is warranted. In the SEER cohort, patients tended to present with more poorly differentiated and advanced stage tumors than those in the Chinese cohort. This is partially because of the differences in race, geographic patterns and diet. International data show that the incidence of small bowel adenocarcinoma in North America, Western Europe and Oceania is higher than that in Asia^12,15^. African Americans have substantially higher incidence rates and worse small bowel adenocarcinoma survival compared to Caucasians^16^. Studies also suggested that dietary factors are related to the risk of small bowel adenocarcinoma^17,18^. High-fat diet led to an increase risk of small bowel tumors in mice. Mechanistically, high-fat diet mediated carcinogenesis may due to DNA damage caused by bile acid. One possible mechanism is that bile acids induce oxidative stress and frequent apoptosis that then causes DNA damage^19^. Other factors, such as alcohol consumption and cigarette smoking, have been suggested to be associated with the risk of small bowel adenocarcinoma^20^. The disparities between the Chinese and SEER cohorts likely reflect a complex interaction between race, geography, environment, socioeconomic and genetic inequalities.

The prognostic factors of resected primary duodenal adenocarcinoma remain controversial. Ryder *et al*. demonstrated that larger tumor size, advanced histological grade, and transmural invasion are associated with decreased survival^10^. Qing-Long Jiang *et al*. revealed that LN metastasis and vascular invasion were independent prognostic factors that were negatively associated with survival in patients undergoing curative resection^21^. In this study, through univariable analysis and subsequent multivariable Cox regression analysis, we identified patients with elder age, worse tumor grade and advanced TNM stage had shorter CSS. Most studies suggested that regional LN metastasis is associated with prognosis^22–24^. The incidence of LN metastasis in patients with duodenal adenocarcinoma has been reported to range from 22% to 76%^25^. Our study, in accordance with previous reports, has shown that patients with nodal metastasis had diminished survival in both the Chinese and SEER cohorts. However, patients in the SEER cohort were approximately twice as likely to be diagnosed as having LN metastasis cancer than those in the Chinese cohort (61.4% vs 30.7%). This may be due to the differences in the T-stage classification between the Chinese cohort and the SEER cohort. T-stage classification was the strongest predictor of LN metastasis in our study, as reported by a matched cohort study based on the National Cancer Database^26^.

The application of nomograms in individualized risk prediction and stratification by incorporating TNM stage and other key prognostic factors is well recognized in a wide variety of cancers, such as prostate, breast, gastric and colorectal cancer^27–30^. In this study, we first constructed a nomogram based on TNM stage along with other clinicopathologic parameters. We found that the TNM-based nomogram predicts CSS more accurately than the AJCC-TNM staging system (C-index value: 0.693 vs 0.625, *P* < 0.001) in the train cohort and 0.677 vs 0.659 (*P* < 0.001) in the Chinese external validation cohort. The calibration plots showed excellent agreement in the training cohort between the prediction probabilities and the actual observations, which ensured the reliability and repeatability of the constructed nomogram. Although there are some differences between the Chinese and SEER cohorts, our nomogram still showed acceptable agreement in the external validation cohort. This nomogram would allow clinicians to identify high risk for poor survival, to make better clinical decisions and provide follow-up surveillance for patients with duodenal adenocarcinoma.

This study has several limitations. Firstly, our retrospective study only included duodenal adenocarcinoma patients received surgical resection, which may result in selection bias. Secondly, variables such as adjuvant chemotherapy and radiation therapy are not available in our study; therefore, some treatment bias is present. Thirdly, the molecular pathologic characteristics are not included in this study, which may result in a limitation on the survival and LN metastasis analysis.

In conclusion, we identified the prognostic factors of duodenal adenocarcinoma patients who underwent curative resection based on two institutions from China and the SEER database. According to the factors, we developed and validated a novel nomogram for predicting postoperative survival of duodenal adenocarcinoma. The nomogram is easy to use, and it provides clear prognostic superiority over the seventh AJCC-TNM staging system. The nomogram might also help clinicians to make individualized predictions of patient survival and to give improved treatment recommendations.

## Materials and Methods

### Patient population

We collected two independent Chinese cohorts that consisted of 137 patients with postoperative duodenal adenocarcinoma at the Second Affiliated Hospital (n = 81) and Sir Run Run Shaw Hospital (n = 56) of Zhejiang University from January 2006 to December 2016. The SEER database was queried for all the patients with postoperative duodenal adenocarcinoma diagnosed between 2004 and 2014. The SEER database is a population-based database sponsored by the National Cancer Institute in the USA that collects cancer incidence and survival data. Available data include patient demographics, primary tumor data, regional nodal data, vital status, and survival. We extracted cases of patients with invasive duodenal adenocarcinoma according to the International Classification of Diseases for Oncology (ICD-O-3), 3^rd^ ed. The program selection codes for the SEER database queries are shown in Supplementary Table S1 and Supplementary Fig. S2. Exclusion criteria include patients with tumors in the ampulla of Vater, pancreas or distal common bile duct; patients without surgery treatment; lack of histology; an indeterminate TNM category; survival for <1 month; and patients with a lifetime occurrence of another primary malignancy. The research protocol of the Chinese cohort was performed in accordance with the guidelines outlined in the Declaration of Helsinki and was approved by the Ethics Committee of the Second Hospital and Sir Run Run Shaw Hospital affiliated with Zhejiang University. All participants informed consent.

### Factors

Parameters included race, age, gender, diagnosis date, tumor size, tumor grade, TNM category, survival status and cancer-specific survival (CSS). We also collected data regarding symptoms, laboratory test results, and vascular and perineural invasion status in the Chinese cohort. The tumor grade was defined as well differentiated, moderately differentiated, poorly differentiated or undifferentiated according to the World Health Organization (WHO) standard grading system. The classification of the depth of invasion, lymph node and distant metastasis was performed according to the AJCC-TNM staging system, 7^th^ ed.

### Follow up

The primary endpoint of this study was CSS, which was registered as the cause-specific classification of death in the SEER database (alive or dead of other cause or cancer-associated death). CSS represents the survival of a specific cause of death in the absence of other causes of death.

### Construction of the nomogram

To construct the effective postoperative CSS nomogram of duodenal adenocarcinoma, we divided the SEER database in two groups randomly. Eighty percent (n = 558) were assigned to the training cohort, and twenty percent (n = 140) were selected as the test cohort. The independent prognostic factors were identified by multivariate Cox proportional hazards regression analysis. Then, a nomogram based on these prognostic factors was constructed by using the train cohort.

### Validation of the nomogram

The prognostic performance of the nomogram was evaluated with discrimination and calibration by using the test and external validation cohort (the independent Chinese patient cohort). Discrimination was assessed with the concordance index (C-index). A higher C-index value indicated a better prognostic accuracy. For calibration, the predicted probabilities produced by the nomogram were compared with the actual probabilities. The Kaplan-Meier method and bootstraps with 1000 resamples were used for this purpose^31^.

### Statistical analysis

Statistical analyses were performed using IBM SPSS version 20.0 and the statistical software package R version 3.4.2. Hazard ratios and their 95% confidence intervals (95% CI) were computed. All P values were 2 sided, and *P* < 0.05 was considered statistically significant.

## Electronic supplementary material

supplementary data
