# Supplementary material for: Prognosis and nomogram for predicting postoperative survival of duodenal adenocarcinoma: A retrospective study in China and the SEER database
Source: Sci Rep. 2018 May 21;8:7940. doi: 10.1038/s41598-018-26145-6 (PMC5962558; doi:10.1038/s41598-018-26145-6)

**Prognosis and nomogram for predicting** **postoperative survival of duodenal adenocarcinoma: A retrospective study in China and the SEER database**

**Running title:** Prognosis of postoperative duodenal adenocarcinoma

Sujing Jiang^1#^, Rongjie Zhao^2#^, Yiran Li^2^, Xufeng Han^3^, Zhen Liu^2^, Weiting Ge^4^, Ying Dong ^1*^, Weidong Han^2*^

^1^Department of Medical Oncology, The Second Affiliated Hospital, College of Medicine, Zhejiang University, Hangzhou, Zhejiang, China; ^2^Department of Medical Oncology, Sir Run Run Shaw Hospital, College of Medicine, Zhejiang University, Hangzhou, Zhejiang, China; ^3^Department of Internal Medicine, Yuyao Traditional Chinese Medicine Hospital, Yuyao, Zhejiang, China; ^4^Cancer Institute, The Second Affiliated Hospital, College of Medicine, Zhejiang University, Hangzhou, Zhejiang, China.

^#^ These authors contributed equally to this work.

***Corresponding authors:**

Weidong Han, Department of Medical Oncology, Sir Run Run Shaw Hospital, College of Medicine, Zhejiang University, 3 East Qingchun Road, Hangzhou 310016, China. Tel: +86 571 86006926; Fax: +86 571 86044817; E-mail: hanwd@zju.edu.cn;

Ying Dong, Department of Medical Oncology, The Second Affiliated Hospital, College of Medicine, Zhejiang University, 88 Jiefang Road, Hangzhou 310009, China. Tel: +86-571-86006922; E-mail: [dongying74@zju.edu.cn](mailto:dongying74@zju.edu.cn).

Supplementary Table S1. The program selection codes for the SEER database

| Variable Name | Variable Description | Position | Length |
| --- | --- | --- | --- |
| PUBCSNUM | Patient ID number | 1 | 8 |
| RACE1V | Race/Ethnicity | 20 | 2 |
| SEX | Sex | 24 | 1 |
| AGE_DX | Age at diagnosis | 25 | 3 |
| YR_BRTH | Year of Birth | 28 | 4 |
| SEQ_NUM | Sequence Number—Central | 35 | 2 |
| YEAR_DX | Year of diagnosis | 39 | 4 |
| PRIMSITE | Primary Site | 43 | 4 |
| HISTO3V | Histologic Type ICD-O-3 | 53 | 4 |
| BEHO3V | Behavior Code ICD-O-3 | 57 | 1 |
| GRADE | Grade | 58 | 1 |
| EOD10_PN | EOD—Extension Prost Path | 69 | 2 |
| EOD10_NE | EOD—Lymph Node Involv | 71 | 2 |
| CSTUMSIZ | CS Tumor Size | 96 | 3 |
| DAJCCT | Derived AJCC T | 128 | 2 |
| DAJCCN | Derived AJCC N | 130 | 2 |
| DAJCCM | Derived AJCC M | 132 | 2 |
| DAJCCSTG | Derived AJCC Stage Group | 134 | 2 |
| SURGPRIF | RX Summ—Surg Prim Site | 159 | 2 |
| SURGSCOF | RX Summ—Scope Reg LN Sur | 161 | 1 |
| SURGSITF | RX Summ—Surg Oth Reg/Dis | 162 | 1 |
| NO_SURG | Reason for no surgery | 166 | 1 |
| REC_NO | SEER Record Number | 176 | 2 |
| TYPE_FU | SEER Type of Follow-up | 191 | 1 |
| SITERWHO | Site Recode ICD-O-3/WHO 2008 | 199 | 5 |
| HISTREC | Histology Recode—Broad Groupings | 226 | 2 |
| cs0204schema | CS Schema v0204+ | 230 | 3 |
| RAC_RECA | Race recode (White, Black, Other) | 233 | 1 |
| STAT_REC | Vital Status recode | 265 | 1 |
| VSRTSADX | SEER Cause-Specific Death Classification | 272 | 1 |
| ODTHCLASS | SEER Other Cause of Death Classification | 273 | 1 |
| CS8SITE | CS Site-Specific Factor 8 | 282 | 1 |
| srv_time_mon | Survival months | 301 | 4 |
| DAJCC7T | Derived AJCC-7 T | 312 | 3 |
| DAJCC7N | Derived AJCC-7 N | 315 | 3 |
| DAJCC7M | Derived AJCC-7 M | 318 | 3 |
| DAJCC7STG | Derived AJCC-7 Stage Grp | 321 | 3 |
| CSMETSDXB_PUB | CS Mets at Dx-Bone | 349 | 1 |
| CSMETSDXBR_PUB | CS Mets at Dx-Brain | 350 | 1 |
| CSMETSDXLIV_PUB | CS Mets at Dx-Liver | 351 | 1 |

Supplementary S2. Flow chart of patient cohort selection in the SEER cohort


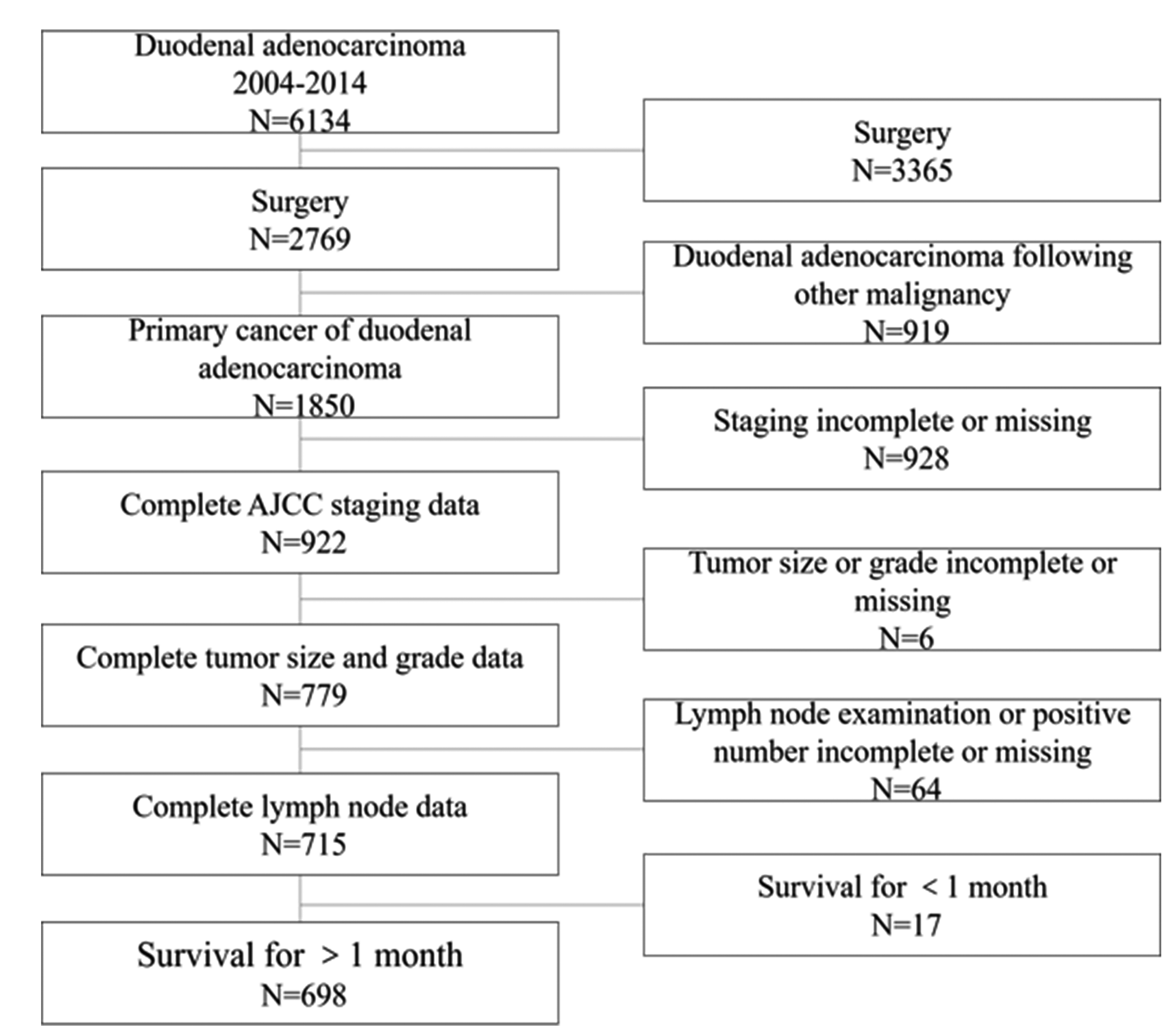

Supplement: Supplementary file 1 — supplementary data [file 41598_2018_26145_MOESM1_ESM.docx]
